# 1511. Sociodemographic and epidemiological factors related to the acceptability of telemedicine among patients for HIV care in four hospitals in the city of Buenos Aires

**DOI:** 10.1093/ofid/ofad500.1346

**Published:** 2023-11-27

**Authors:** Maria C Acosta, Tomas Kierszenowicz, Maria Cecilia Acosta, Maria Jose Rolon, Mercedes Cabrini, Diego Cecchini, Claudia Gabriela Rodriguez, Pablo Scapellato, Edgardo Gabriel Bottaro, Marcelo H Losso

**Affiliations:** Hospital J.M. Ramos Mejía, Buenos Aires City, Ciudad Autonoma de Buenos Aires, Argentina; Consejo Nacional de Investigaciones Científicas y Técnicas, Buenos Aires, Ciudad Autonoma de Buenos Aires, Argentina; Hospital J.M. Ramos Mejía, Buenos Aires City, Ciudad Autonoma de Buenos Aires, Argentina; Hospital Dr. Juan A. Fernández, Buenos Aires, Ciudad Autonoma de Buenos Aires, Argentina; Hospital Dr. Juan A. Fernández, Buenos Aires, Ciudad Autonoma de Buenos Aires, Argentina; Hospital Dr. Cosme Argerich, Buenos Aires, Ciudad Autonoma de Buenos Aires, Argentina; Hospital Dr. Cosme Argerich, Buenos Aires, Ciudad Autonoma de Buenos Aires, Argentina; Hospital Donación Francisco Santojanni, Ciudad Autonoma de Buenos Aires, Ciudad Autonoma de Buenos Aires, Argentina; Hospital Donación F. Santojanni., Buenos Aires, Ciudad Autonoma de Buenos Aires, Argentina; Hospital JM Ramos Mejía, Buenos Aires, Ciudad Autonoma de Buenos Aires, Argentina

## Abstract

**Background:**

In October 2020, a research consortium by four HIV and infectious diseases units of general acute public hospitals of Buenos Aires city began an implementation study aimed to analyze obstacles and facilitators of telemedicine (TM) in the care of people living with HIV(PLHIV). TM visits were conducted through phone or videocalls

**Methods:**

This research aims to assess the association of sociodemographic and epidemiological factors and the willingness of HIV patients to continue with TM after the sequelae of COVID-19. We prospectively collected quantitative data from medical records and distributed an anonymous semi-structured survey to participants after their first TM visit, collecting data on willingness to continue with this strategy and sociodemographic characteristics. We included all the participants who answered the survey. The data was stored in the Redcap® platform. Continuous data are shown as medians with interquartile range (IQR) and were compared using the Wilcoxon test. Categorical data are described as percentages and compared using the chi-square test. We use R® software

**Results:**

The survey was delivered to 4118 patients who had their first remote visit between October 2020 and October 2022. 1.871 patients (45%) responded the survey. 1.085 (58%) were cisgender men. The median age was 47 years (IQR: 37-54). 1.221 (65%) would like to continue with this strategy, while 618 (33%) would not. 32 (2%) did not respond. 1102 (90%) patients willing to continue TM had wireless access at home and 544 (88%) unwilling to continue had wireless access. Among those who were willing to continue using TM, the proportion of people with viral suppression was 88.6%, while among those who were not willing to continue using TM, the proportion was 87.1%. The median CD4 count was 639 (IQR: 442.5-907) for those who wanted to continue and 657 (423-917) for those who did not

**Conclusion:**

In this adult cohort of PLHIV receiving TM care in public facilities in the city of Buenos Aires, two-thirds of those who answered the survey were willing to continue with this strategy after the COVID-19 crisis. When comparing participants willing or unwilling to continue TM in the future, both groups were similar in terms of age, gender, proportion of participants with wireless access at home, CD4 count and viral suppression

**Disclosures:**

**All Authors**: No reported disclosures

